# The Role of Self-Compassion in Buffering Symptoms of Depression in the General Population

**DOI:** 10.1371/journal.pone.0136598

**Published:** 2015-10-02

**Authors:** Annett Körner, Adina Coroiu, Laura Copeland, Carlos Gomez-Garibello, Cornelia Albani, Markus Zenger, Elmar Brähler

**Affiliations:** 1 Department of Educational and Counselling Psychology, McGill University, Montreal, Canada; 2 Department of Oncology, McGill University, Montreal, Canada; 3 Louise-Granofsky-Psychosocial Oncology Program, Segal Cancer Centre, Jewish General Hospital, Montreal, Canada; 4 Psychosocial Oncology Program, McGill University Health Centre, Montreal, Canada; 5 University of Leipzig, Department of Medical Psychology and Medical Sociology, Leipzig, Germany; 6 Faculty of Applied Human Studies, University of Applied Sciences Magdeburg and Stendal, Stendal, Germany; 7 Department of Psychosomatic Medicine and Psychotherapy, Universal Medical Center Mainz, Mainz, Germany; University of Stellenbosch, SOUTH AFRICA

## Abstract

Self-compassion, typically operationalized as the total score of the Self-Compassion Scale (SCS; Neff, 2003b), has been shown to be related to increased psychological well-being and lower depression in students of the social sciences, users of psychology websites and psychotherapy patients. The current study builds on the existing literature by examining the link between self-compassion and depressive symptomatology in a sample representative of the German general population (n = 2,404). The SCS subscales of self-judgment, isolation, and over-identification, and the “self-coldness”, composite score, which encompass these three negative subscales, consistently differed between subsamples of individuals without any depressive symptoms, with any depressive syndromes, and with major depressive disorder. The contribution of the positive SCS subscales of self-kindness, common humanity, and mindfulness to the variance in depressive symptomatology was almost negligible. However, when combined to a “self-compassion composite”, the positive SCS subscales significantly moderated the relationship between “self-coldness” and depressive symptoms in the general population. This speaks for self-compassion having the potential to buffer self-coldness related to depression—providing an argument for interventions that foster self-caring, kind, and forgiving attitudes towards oneself.

## Introduction

Depression is a long lasting, often recurring mood disorder and one of the most frequent and debilitating mental health issues today [1–4]. It is estimated that more than 350 million people worldwide suffer from depression [5]. Depressive disorders cause immense personal suffering and pose a serious economic problem: as a chronic-type of condition, depression often requires repeated cycles of treatment, which are demanding to the health care system [4, 6, 7]. Depression is the 4th leading cause of disability worldwide, resulting in time away from work [8]. Further, major depressive disorder has been associated with decreased functioning in major life areas, such as marriage, parenting, and career [9]. Given the personal and societal burden incurred by depressive disorders, it is crucial that effective strategies for preventing depressive symptoms are investigated and tested. For example, self-compassion, a relatively new construct in the psychological literature, has been shown to negatively relate to depressive symptomatology [10–15]. This link is worth investigating further, as psychological interventions aiming to enhance self-compassion might be effective at curbing the rates of depression that are currently seen worldwide [16–18].

Self-compassion has been defined as a healthy attitude towards oneself during times of struggle [10]. It differs from other self-concepts, such as self-esteem [19, 20], which is a reflection of external standards upon one’s individual self-worth. With self—compassion, the focus is less on individual characteristics and more on what is common across all members of the humankind [21, 22]. As per its main theorist [21], who drew upon Buddhist philosophy, self-compassion encompasses three key dimensions, which are each defined through two opposite poles: a) the presence of self-kindness in the absence of self-judgment; b) a sense of common humanity as opposed to a sense of isolation; and c) mindfulness as opposed to over-identification. *Self-kindness* refers to being caring, understanding and accepting of oneself, becoming aware of, and being moved by one’s own suffering whereas self-judgment involves being harsh and extremely self-critical (Neff, 2003a). Gilbert and Irons (2005) proposed that in order for one to experience self-kindness, one has to develop self-awareness of the detrimental effect of self-judgment. As such, the two extreme attitudes can be situated on the same continuum. *Common humanity* helps one recognize that failure and hardship are a shared human experience rather than something that sets one apart from others. This fosters a feeling of connectedness to others rather than leaving one feeling isolated and alone when in pain or suffering. *Mindfulness* represents the profound acknowledgment and acceptance of the present moment experience, neither avoiding, nor ruminating on painful emotions, but trying to balance them. It involves taking more of an objective stance on one’s experience in order to gain perspective and to avoid over-identification with negative thoughts and emotions [21]. Self-kindness, common humanity, and mindfulness contribute to what Neff [23] labeled as a “self-compassionate frame-of-mind”. Similarly, Gilbert, McEwan [24] used the term “self-compassion”to refer to a composite of the three positive components of Neff’s self-compassion scale while naming the composite captured by the three negative subscales, i.e., self-judgment, isolation, and over-identification, “self-coldness”.

Self-compassion (SCS) as a construct composed of both positive and negative aspects, has been found to relate positively to positive affect and negatively to negative affect [25–27]. Further, it is positively associated to optimism, happiness, personal initiative, openness to experience, and well-being [12, 26–28]. A meta-analysis corroborated these findings linking higher self-compassion to lower levels of psychological stress, anxiety and depressive symptoms [29]. The positive aspects of self-compassion have been negatively associated with depressive symptoms among student [13, 14, 33] and community samples of individuals undergoing treatment or seeking mental health services for depression and/or anxiety [30, 31]. The negative aspects of self-compassion, i.e., self-judgment, isolation and over-identification, have shown positive associations with symptoms of depression and loneliness in patients [30, 31], student populations [13, 32] and community samples [14, 28]. Notably, the relationships between depression and the negative subscales of the SCS were generally stronger than those between depression and the positive subscales of SCS [13, 28, 30, 31]. The majority of these studies were conducted with social sciences students [13, 14, 33] and other individuals previously exposed to psychological content such as psychology-related college courses [31], psychology online-research [28], psychotherapy [29], or other mental health services [30]. Arguably these samples constitute a segment of the population, which is likely aware of the benefits of self-care and of psychological interventions to alleviate distress. As such, it is reasonable to expect that they differ from the general population with respect to their grasp of the concept of self-compassion, their psychological awareness, and potentially even their levels of self-compassion.

The current level of evidence points toward an association between depression and self-compassion in student, community, and clinical samples. To our knowledge, no study has examined the relationship between the positive and negative aspects of self-compassion (as per [10, 21]) and depression in the general population.

### Study Objective and Rationale

The present study aims to extend the existing literature by drawing inferences about self-compassion, self-coldness, and depressive symptomatology. Specifically, this study will 1) examine the levels of self-compassion (positive self-compassion and self-coldness) across three different sub-groups of individuals with varying levels of depressive symptomatology; 2) determine which self-compassion subconstructs are the strongest predictors of depressive symptoms; 3) test the moderating effect of self-compassion (i.e., the positive aspects of self-compassion) on the relationship between self-coldness (defined as the negative aspects of a self-compassion scale) and depressive symptoms in a large sample recruited from the general population Germany’s. It is hypothesized that self-compassion levels will be different across subgroups of individuals without depressive symptoms, with any depressive syndromes, and with major depression disorder. No a priori hypothesis has been stated for the research question #2. Finally, it is expected that self-compassion will moderate the relationship between self-coldness and depression.

## Method

### Participants and Recruitment Procedure

#### Ethics statement

The present study is based on a nation-wide survey conducted in 2012 with a representative sample of Germany’s general population. A research assistant presented all eligible participants with an overview of the study procedures, delivered a detailed data privacy statement, and informed the potential participant about the anonymization of all personal data. Subsequently, the research assistant obtained verbal informed consent, which was noted in the study documents before the survey was administered. The study posed a minimal risk to participants as the study did not involve any medical treatments, invasive diagnostic procedures, or procedures that could cause psychological, spiritual, or social harm. Therefore, according to German law, verbal consent was considered appropriate and an additional informed consent of a parent or guardian was not required for participants aged 14 years or older. The ethics board of the University of Leipzig, Germany (Ethics protocol # 092–12–05032012) granted ethical approval of the study protocol including the consent procedures. Furthermore, the study adhered to the ethical principles of the *International Code of Marketing and Social Research Practice* by the International Chamber of Commerce (ICC) and the European Society for Opinion and Marketing Research (ESOMAR).

#### Data collection

The data were collected by the Independent Service for Surveys Methods and Analyses, USUMA GmbH, using the random-route-technique and the Kish selection grid [34, 35]. Study representatives made a total of four attempts, including the initial contact, with all eligible participants. Eligibility included the ability to read and write in German and being over the age of 14. Across 320 geographical sampling points, 4,480 households were selected to participate in the survey, of which 44 were not eligible (e.g., vacant home). Participation rate was 56.5%. Trained interviewers recorded the socio-demographic information from consenting participants (*n* = 2,510), who completed the self-report questionnaires anonymously. The current study includes 2,404 participants who were older than 17 years and had no missing answers on the outcome measure, i.e., the Patient Health Questionnaire [36]. In comparison to the data available from the German Census Bureau [37], the current study has a slightly higher proportion of women (53.7% in the study sample versus 50.9% in the census population). Socio-demographic characteristics of the study sample are included in Table 1.

**Table 1:** Socio-demographic Characteristics of the Study Sample (n = 2,404)

Variable	Total	Men	Women
	*n* = 2,404	*n* = 1,114	*n* = 1,290
Age in years			
M *(SD*)	50.19 (17.36)	50.09 (17.32)	50.28 (17.40)
Range	18–91	18–91	18–91
	**N (%)**	**N (%)**	**N (%)**
Age groups in years			
18–29	390 (16.2)	187 (16.8)	203 (15.7)
30–39	339 (14.1)	158 (14.2)	181 (14.0)
40–49	390 (16.2)	172 (15.4)	218 (16.9)
50–59	493 (20.5)	223 (20.0)	270 (20.9)
60–69	407 (16.9)	195 (17.5)	212 (16.4)
70 and above	385 (16.0)	179 (16.1)	206 (16.0)
Relationship status			
Married and/or cohabitation	1,228 (51.1)	619 (55.6)	609 (47.2)
Married, but separated	35 (1.5)	14 (1.3)	21 (1.6)
Divorced	293 (12.2)	108 (9.7)	185 (14.3)
Widowed	276 (11.5)	70 (6.3)	206 (16.0)
Single, never married	572 (23.8)	303 (27.2)	269 (20.9)
Education level completed			
≤ 8 years	960 (39.9)	442 (39.6)	518 (40.1)
9–11 years	1,018 (42.4)	436 (39.1)	582 (45.2)
≥ 12 years	415 (17.3)	232 (20.9)	183 (14.2)
Student	11 (0.4)	4 (0.4)	7 (0.5)
Employment status			
Full time	1,016 (42.2)	613 (55.0)	403 (31.2)
Part-time	313 (13.0)	30 (2.7)	283 (21.9)
Military/civilian service; maternity leave	16 (0.7)	1 (0.1)	15 (1.2)
Homemaker	110 (4.6)	10 (0.9)	100 (7.8)
Retired	712 (29.6)	343 (30.8)	369 (28.6)
In training	136 (5.7)	65 (5.8)	71 (5.5)
Unemployed	101 (4.2)	52 (4.7)	49 (3.8)

### Measures

#### Depressive symptoms

The severity of depressive symptomatology was assessed via the Patient Health Questionnaire [36]. The PHQ-9 is the depression module of the Patient Health Questionnaire [38], which is an integral part of a more comprehensive diagnostic tool, the Primary Care Evaluation of Mental Disorders (PRIME-MD). The PHQ-9 is a brief self-administered screening tool intended to aid clinicians to diagnose depressive disorders and their severity based on the DSM-IV criteria. It asks respondents to indicate how often during the previous two weeks they were bothered by problems such as “feeling down, depressed, or hopeless”. The PHQ-9 items are rated on a 4-point Likert-type scale ranging from 0 (*not at all*) to 3 (*nearly every day*), with higher scores indicating more symptoms and higher severity. A diagnosis of *major depression disorder* (MDD) is probable if at least 5 out of the 9 symptoms are present “more than half of the days” (≥ 2) *and* one of the symptoms endorsed is depressed mood or anhedonia. Further, if the item about suicidal ideation (i.e., “thoughts that you would be better off dead or of hurting yourself in some way”) is endorsed at any level, it is counted toward the score for MDD [39]. The presence of *other depressive symptoms* is probable if 2 to 4 symptoms are endorsed as being present at “more than half of the days” (the suicidal ideation item is counted if endorsed at all) *and* one of the symptoms endorsed is depressed mood or anhedonia [36]. The PHQ-9 was initially validated with a sample of 6000 patients attending primary health care and obstetrics and gynecology clinics and it has shown good construct validity (via associations with measures assessing functional status, days off taken due to disability, and symptom-related problems) and criterion validity (via associations between PHQ-9 scores and findings from two clinical interviews) [36]. Evidence for the scale’s construct validity within the general population is based on studies conducted in 2003 and 2008 among the German general population with a total of 5,018 participants with ages between 14 and 92 years [40]. Further, the internal consistency of the tool was α = .89 in the primary health care sample,. 86 in the gynecology sample,. 87 in the general population sample of Kocalevent, Hinz (40), and. 86 in the current study sample (*M* = 2.29, *SD* = 3.21). The 48-hour test-retest reliability was. 84 [36]. The construct validity of the German version of the PHQ-9 was established in a large community sample (*n* = 2060) representative of the general population via associations with measures of depression, quality of life, metal health status, and symptom checklists [41]. In the current study, the PHQ-9 total score and composite scores corresponding to probable diagnoses of MDD and other depressive syndromes were used in the analyses. The internal consistency reliability for the current sample (Cronbach’s alpha = .86) has been reported elsewhere [alpha = .86; 42]).

#### Self-compassion

Self-compassion was assessed using the German version of the Self Compassion Scale [10, 28]. The SCS is a 26-item self-report questionnaire assessing ways of relating to oneself via six subscales. The three subscales representing positive self-compassion attitudes, i.e., self-kindness (sample item, “When I’m going through a very hard time, I give myself the caring and tenderness I need”), common humanity (sample item, “When I feel inadequate in some way, I try to remind myself that feelings of inadequacy are shared by most people”), and mindfulness (sample item, “When something upsets me, I try to keep my emotions in balance”) are phrased as positive statements. The negative self-compassion subscales, i.e., self-judgment (sample item, “I’m disapproving and judgmental about my own flaws and inadequacies”), isolation (sample item, “When I fail at something that’s important to me, I tend to feel alone in my failure”), and over-identification (sample item, “When I’m feeling down I tend to obsess and fixate on everything that’s wrong”) are phrased in a negative direction and reflect a lack of self-compassion [10, 21]. Items are scored on a 5-point Likert-type scale ranging from 1(*very rarely*) to 5 (*very often*), with higher scores indicating higher levels of the construct measured. In the current sample, reliability coefficients ranged from α = .70 (over-identification) to. 79 (self-kindness). A total SCS score is obtained by computing the mean across all 26 items after reverse scoring the negative subscale items. As per previous reports [13, 24], two composite scores were also computed. The *self-compassion composite* score was computed as the mean across the 13 items of the three positive SCS subscales (α = .89). The composite score for *self-coldness* was computed as mean across the 13 items of the three negative SCS subscales (α = .87).

In the development sample comprising of undergraduate students from the United States, the internal consistency for the total scale was α = .92 and ranged from. 75 to. 81 for the subscales [10] [10]. Cronbach’s alpha for the self-compassion and the self-coldness composite scores ranged between. 89 and. 93 in samples of students and psychotherapists [24]. The three-week test-retest reliability for the total scale was. 92 and ranged from. 80 to. 88 for the subscales [10]. Convergent and divergent validity of the SCS was established via positive associations with measures assessing social connectedness, life satisfaction, self-esteem, and emotional processing and via negative associations with measures of distress (e.g., anxiety and depression), self-criticism, neurotic perfectionism, and rumination [10].

### Data Analysis

Descriptive and inference statistics (i.e., means, standard deviations, and comparisons across subgroups of individuals with no depressive symptoms, probable MDD, and other depressive syndromes) were computed for the self-compassion scales. In order to reduce the probability of Type I errors, a Bonferroni correction was applied to the group comparisons via t-tests (i.e., requiring *p* < 0.002 to establish statistical significance).

A regression model including the PHQ-9 scores as the dependent variable (DV), and the six self-compassion subscales as independent variables (IV) was computed. Due to the exploratory nature of this analysis, the method employed to enter the predictors was stepwise. Age, sex and educational attainment were added as covariates due to having been related to depression and/or self-compassion in previous studies. In addition, a moderation model was tested as follows: the self-coldness composite was conceptualized as the IV; the PHQ-9 was entered as the DV, and the self-compassion composite score as the moderator. The statistical analyses were conducted in SPSS 21; the moderation analysis was run using the PROCESS macro developed by Preacher and Hayes [20, 43], which was imported into SPSS 21.

## Results

Descriptive and inferential statistics for the self-compassion scale (means, standard deviations, and comparisons between three subgroups of the total study sample, i.e., individuals with no depressive symptoms (*n* = 959), with any depressive syndromes but not major depressive disorder [MDD] (*n* = 78), and with symptoms consistent with a diagnosis of MDD (*n* = 50) are included in Table 2. In the subgroup of individuals with a probable MDD, the mean (M) and standard deviation (SD) for the PHQ-9 were 15.29 and 3.02, with scores ranging from 11–26. Among individuals with the likelihood of having another depressive syndrome than MDD, the PHQ scores were *M* = 8.76, *SD* = 2.54, range = 3–16. Regardless of whether the Bonferroni was applied or not, increased depressive symptomatology was associated with lower scores on the positive SCS subscales, the self-compassion composite, and the SCS total; further, it was also associated with higher scores on the negative SCS subscales and the self-coldness composite. More specifically, compared to the subsample of persons with no depressive symptoms, those with any depressive syndrome or with MDD reported lower self-kindness and mindfulness, and more isolation, self-judgment and over-identification. The self-compassion composite score was comparable across those with no depressive symptoms and those with any depressive syndromes. However, the MDD group scored significantly lower than the non-depressive group. Self-coldness was significantly higher in the MDD group than in the other two groups. Further, the scores of all three negative SCS subscales differed significantly between all three subsamples, with highest scores in the MDD group and lowest in the group without any symptoms of depression.

**Table 2:** Differences in SCS Subscale, Total and Composite Scores between Subsamples with Varying Degrees of Depressive Symptomatology (n = 1,078)

Variable (Cronbach’s α)	No depressive symptoms (*n*_*a*_ = 959)	Other depressive syndrome^#^ (*n*_*b*_ = 78)	Major depressive disorder (*n*_*c*_ = 50)	*t*_(a-b)_	*t*_(a-c)_	*t*_(b-c)_
SCS Self-kindness (α = .79)	3.01 (0.82)	2.72 (0.82)	2.74 (0.73)	3.00*	2.28*	ns
SCS Common humanity (α = .73)	2.88 (0.89)	2.97 (0.70)	2.70 (0.75)	ns	ns	ns
SCS Mindfulness (α = .77)	3.12 (0.87)	3.02 (0.62)	2.72 (0.79)	ns	3.18**	2.40*
SCS Self-judgment (α = .73)	2.10 (0.68)	2.77 (0.74)	3.07 (0.83)	8.31**	9.72**	2.13*
SCS Isolation (α = .77)	1.99 (0.73)	3.05 (0.81)	3.40 (0.86)	12.23**	13.19**	2.33*
SCS Over-identification (α = .70)	2.08 (0.70)	2.95 (0.67)	3.22 (0.77)	10.59**	11.17**	2.10*
SCS Total Scale (α = .88)	3.47 (0.40)	3.02 (0.46)	2.74 (0.48)	9.44**	12.45**	3.30**
Self-compassion composite (α = .88)	3.00 (0.76)	2.90 (0.60)	2.72 (0.62)	ns	2.56*	ns
Self-coldness composite (α = .87)	2.06 (0.60)	2.91 (0.60)	3.22 (0.66)	12.03**	13.26**	2.74*

*Note*.

^#^ Subgroup of individuals, whose PHQ-9 data indicate a high probability of the presence of another depressive syndrome than major depressive disorder

SCS = Self-Compassion Scale; ns = non significant

*p < .05 without correction

**p < .002 as per Bonferroni correction.

### Predictors of Depressive Symptomatology

A step-wise multiple regression predicting PHQ-9 scores from SCS subscales while controlling for the effect of age, sex, and education was conducted. Tests to see if the data met the assumption of collinearity indicated that multicollinearity was not a concern, Tolerance *Range* = .51-.69, variance inflation factor [VIF] *Range* = 1.00–1.95 [44]. This analysis showed that a total of 23% of the variance in depressive symptom severity was explained by the SCS, overall *F*(8, 2310) = 101.00, *p* < .001 (coefficients displayed in Table 3). Specifically, isolation accounted for 18% of the variance in symptom severity; over-identification accounted for an additional 2%; lack of self-kindness accounted for 2% of the variance; self-judgment and lack of mindfulness accounted for less than 1% each. Common humanity was not significantly associated with depression and was excluded from all six models of the regression analysis.

**Table 3:** Prediction of Depressive Symptomatology (PHQ-9) in the General Population by Self-Compassion Subscales (n = 2,404).

Variable	*R* ^2^	Δ*R* ^2^	Δ*F*	β
Model 1—Covariates	.03	.03	20.40*	
Age				.16*
Sex				.03
Education				.01
Model 2	.21	.18	538.24*	
SCS Isolation				.43*
Model 3	.23	.02	59.89*	
SCS Isolation				.30*
SCS Over-identification				.19*
Model 4	.25	.02	51.33*	
SCS Isolation				.30*
SCS Over-identification				.22*
SCS Kindness				-.13*
Model 5	.25	.01	22.19*	
SCS Isolation				.27*
SCS Over-identification				.16*
SCS Kindness				-.13*
SCS Judgment				.11*
Model 6	.26	.01	17.55*	
SCS Isolation				.26*
SCS Over-identification				.16*
SCS Kindness				-.07*
SCS Judgment				.14*
SCS Mindfulness				-.10*
Overall *F*(8, 2310) = 101.00*				

*Note*. Age, biological sex, and level of education were controlled for in all models. PHQ-9 = Patient Health Questionnaire; SCS = Self-Compassion Scale.

*p < .001.

### Self-Compassion as a Protective Factor against Depression

**Fig 1:**
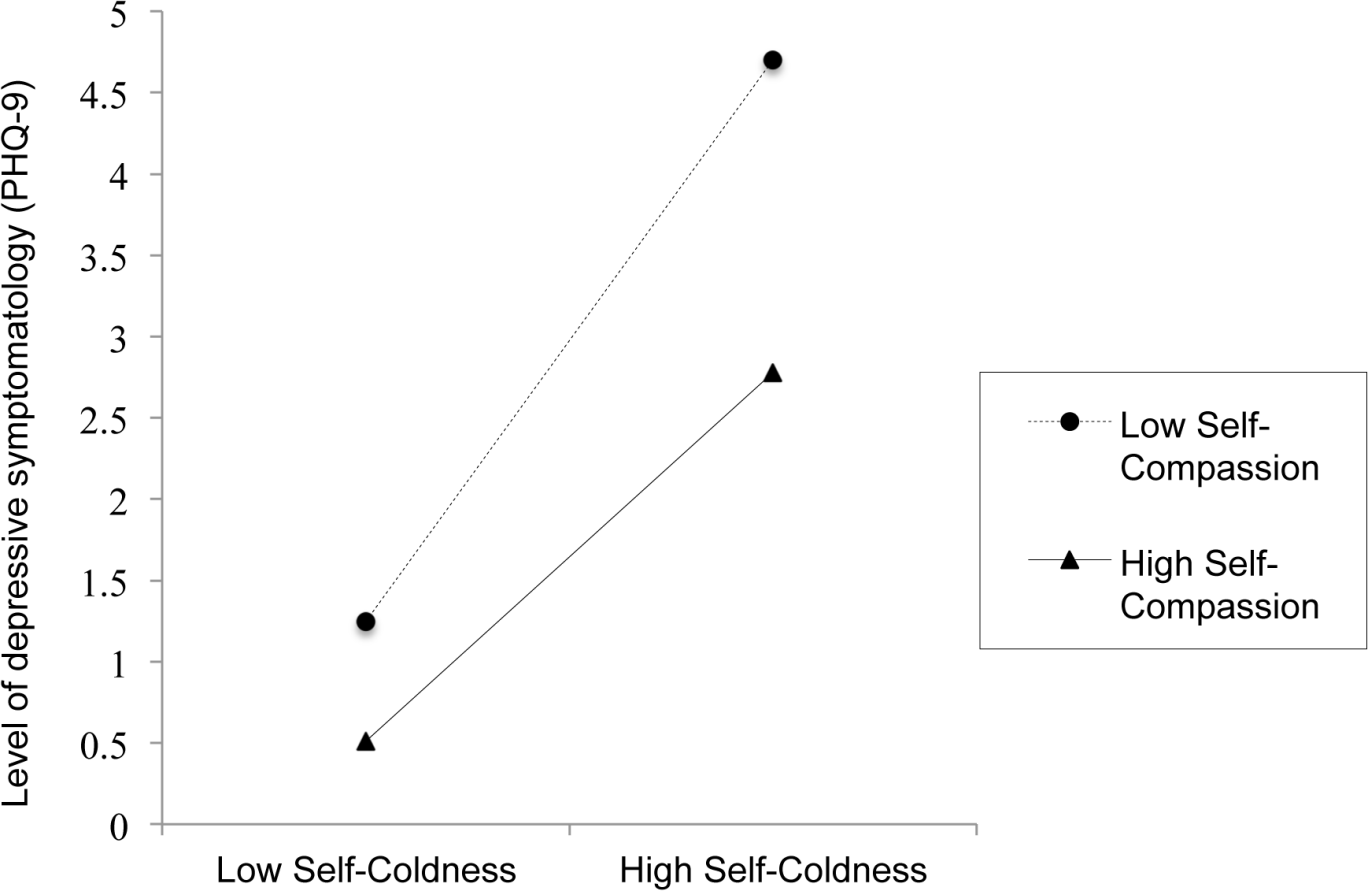
Illustration of the moderation of the relationship between self-coldness and depression by self-compassion (n = 2,404).

Moderation was tested via methodology developed by Preacher and Hayes [45]. The hypothesized model suggested a significant interaction effect of self-compassion and self-coldness on depression scores (*p* < .001). The fact that the lower and upper limits of the 95% confidence interval (CI) for the interaction did not cross the zero mark, further corroborates the statistical significance of the moderation effect (see Table 4 for detailed results). As such, this model was considered significant, *R* ^2^ = .23, *MSE* = .09, *F*(3, 2307) = 229.07, *p* < .001. The variance increase due to the interaction effect was *R* ^2^ = .01. The moderation effect is plotted in Fig 1. As the relationship between self-coldness and depression severity was moderated by self-compassion at all levels (i.e., high, average, low) of the moderator, a slope analysis was conducted to better understand this moderation effect. Following guidelines developed by Hayes [46], a simple slope analysis assessed the relationship between self-coldness and depression at high (i.e., 1 SD above the mean) and low (i.e., 1 SD below the mean) levels of the moderator (i.e., positive SCS). The association between self-coldness and depression was significantly weaker (*t*(2312) = -.6.75, *p* < .001) among individuals with high self-compassion (β = 1.76, *t* = 9.24, *p* < .001) than among those with low self-compassion (β = 2.69, *t* = 16.53, *p* < .001).

**Table 4:** Moderation of the Relationship Between Self-Coldness and PHQ-9 by Self-Compassion (n = 2,311)

Variable	*B*	*SE*	*t*	*p*	95% CI
SCS Self-Compassion Composite (COMPASSION)	-0.11	0.01	-9.95	< .001	[-0.13, -0.09]
SCS Self-Coldness Composite (COLDNESS)	0.25	0.01	23.65	< .001	[0.23, 0.27]
COMPASSION X COLDNESS	-0.08	0.01	-5.44	< .001	[-0.10, -0.05]

*Note*. SCS = Self-Compassion Scale; PHQ-9 = Patient Health Questionnaire.

### Exploratory Analyses: A Second Moderation Model

Given the significant moderation effect in the direction it was expected and based on the results of the regression analysis, a second unplanned, but more specific moderation model was tested with isolation as the IV, PHQ-9 as the DV, and self-kindness as the moderator. The model assessing the moderation effect of isolation and self-kindness upon depression scores was also significant, *R* ^2^ = .21, *MSE* = .10, *F*(3, 2358) = 208.92, *p* < .001. The variance increase due to the interaction effect was *R* ^2^ = .01. The significance of the moderation effect was corroborated by the fact that the 95% CIs did not cross the zero value. The detailed results of this moderation analysis are presented in Table 5.

**Table 5:** Moderation of the Relationship Between Isolation and PHQ-9 by Self-Kindness (n = 2,362)

Variable	*B*	*SE*	*t*	*p*	95% CI
SCS Self-Kindness	-0.07	0.01	-7.90	< .001	[-0.09, -0.05]
SCS Isolation	.19	0.01	22.71	< .001	[0.17, 0.21]
SCS Self-Kindness X SCS Isolation	-0.06	0.01	-6.07	< .001	[-0.08, -0.04]

*Note*. SCS = Self-Compassion Scale; PHQ-9 = Patient Health Questionnaire.

## Discussion

The current study examined the levels of self-compassion in three groups with varying levels of depressive symptomatology, the relationship between self-compassion and depressive symptomatology in the general population as well as the buffering effect of self-compassion upon depressive symptomatology. Self-judgment, isolation, and over-identification differed significantly between subgroups of non-depressed individuals and individuals with a probable depressive disorder. As the depressive symptomatology increased as a function of group, the self-coldness, or the lack of self-compassion, also increased. This finding is in line with the scholarship to date [28, 30, 31]. The findings for the positive subscales of the SCS, i.e., self-compassion composite, were less consistent and weaker in their capacity to differentiate between groups of individuals with more or less depressive symptomatology. As anticipated, self-kindness and mindfulness differentiated between the group of individuals without depressive symptoms and those with a probable major depressive disorder (MDD). Also, the means of all three positive subscales in our subgroup with probable MDD closely resemble the scores reported by Krieger, Altenstein (31) for 114 German patients with moderate to severe depression (*M*_*self-kindness*_ = 2.47, *M*_*common humanity*_ = 2.55, *M*_*mindfulness*_ = 2.82). Their severely depressed group reported more self-judgment (*M* = 3.55) and over-identification (*M* = 3.62) than our participants in the MDD group. Yet, this difference may be explained by the fact that all participants of Krieger, Altenstein (31) suffered from depression to the extent that they sought treatment and were accepted into a clinical trial while not all our subjects with a probable diagnosis of major depression based on the PHQ-9 self-report screener may indeed suffer from clinical depression [36, 38].

This study also intended to identify the components of Neff’s construct of self-compassion that are the strongest predictors of depressive symptoms in the general population. Isolation predicted the most variance in depression scores, which is consistent with the associations of this construct among populations previously exposed to psychological constructs [13, 47] and patient populations [30, 31]. Surprisingly, self-judgment and over-identification predicted only little variance in symptom severity—contrasting a study with 504 anxious and/or depressed patients for whom self-judgment explained 36% of the severity of their depressive symptomatology [30]. However, in the latter sample over-identification also explained less than 1% of the variance. As such it seems that within our general population sample of which the vast majority did not report clinical depressive symptoms, being self-judgmental was a lower risk factor for depression than perceiving oneself as isolated and alone in one’s struggles and inadequacies. This is in line with the well-established link between depression and the thwarted need for relatedness/ sense of belonging [48, 49]. It was not surprising to find that common humanity did not relate to depression: similarly, no relationship was found between common humanity and depression in samples of depressed patients and never-depressed individuals from the community [31]; in college samples this relationship was weak, i.e., r < .20 [13, 33]; common humanity showed weaker associations with depression compared to the other self-compassion subscales in students, clinical and community samples [13, 28, 30, 33, 47]. It was, however, surprising that self-kindness and mindfulness had a negligible contribution to the variance in depression scores in our sample. Previous self-compassion research has consistently shown a positive relationship between self-kindness and depression—albeit with varying magnitudes and highest scores being reported among student samples [13, 28, 31, 33, 47].

In line with the literature, our data show that self-compassion (i.e., a composite score based on the positive SCS subscales self-kindness, common humanity, and mindfulness) moderates the effect of self-coldness (i.e., self-judgment, isolation, and over-identification) on depressive symptoms. This suggests that self-compassion is a protective factor against depression, particularly among individuals who experience high self-coldness. In order to better understand the interaction effect between self-compassion and self-coldness upon depressive symptomatology and to contextualize the findings from the regression analysis, a second moderation model, which was not planned a priori, was tested at SCS subscale level. The significant moderation effect of self-kindness on the relationship between isolation and depression suggests that within the general population a) it might be isolation that highly associates with depression, and b) that self-kindness can attenuate the link between isolation and depression. These findings are in line with [33] who demonstrated that self-kindness moderates the relationship between depression and a composite measure of “perfectionism, control needs, and defensive separation”. However, it is noteworthy that in the current study only a small moderation effect (i.e., 1% of variance explained by the interaction effect) was detected for both self-compassion composite score and self-kindness. As such, the potentially protective effect of self-compassionate attitudes towards oneself needs to be corroborated by further research in order to determine its clinical relevance and to clarify the psychological pathways of the widely assumed benefits of self-compassion for psychological wellbeing in the general population.

Empirical evidence regarding the relationship between depression and self-compassion guided the analyses employed in the current study. The large sample size and the rigorous recruitment strategy, provide confidence that the results are highly applicable to the general population of German speakers. Notwithstanding the strengths of this study, there are several considerations that need to be discussed when interpreting these findings. Given the cross-sectional design, the directionality of our results could not be established. Further, our findings are highly dependent on the measurement tools. While the PHQ-9 has been validated thoroughly in clinical and general population samples, it cannot provide a clinical diagnosis: at best, it screens for probable depressive disorders. The SCS, while extensively validated in populations such as students of the social sciences, users of psychology websites and psychotherapy patients, would benefit from future investigations into its utility and validity for the general population. Individuals with lower educational attainment and less or no prior exposure to psychological concepts may have more difficulty with phrasings such as “I try to be loving towards myself …” or “I approach my feelings with curiosity and openness” [22, 50]. Future studies should further investigate the link between self-compassion, self-coldness and depression to gain a better understanding of the potentially beneficial effects of self-compassion, particularly among individuals vulnerable to depression. As self-compassion can be fostered via psychotherapy [17, 18, 51, 52], workshops [16], online-interventions [53] and self-help work [54] that promote self-compassion could be a feasible avenue for preventing depression in adults who are prone to increased self-judgment, isolation and/or over-identification. Based on findings in youth samples regarding the association of self-compassion and childhood maltreatment, later emotion regulation difficulties, and adolescent mental health [55–59] one could envision prevention campaigns focused on increasing self-compassion, fostering resilience and promoting well-being among youth.

The current study provides a first glimpse at the relevance of the self-compassion construct at the level of the general population. Our core finding that self-compassion has the potential to buffer the relationship between self-coldness and depression provides an argument for future research into beneficial effects of self-compassion for psychological wellbeing in the general population.
