# Correction: The Role of Self-Compassion in Buffering Symptoms of Depression in the General Population

**DOI:** 10.1371/journal.pone.0142027

**Published:** 2015-10-30

**Authors:** The PLOS ONE Staff

There are errors in the author affiliations in the XML version of the article. The publisher apologizes for these errors. The affiliations should appear as shown here:

Annett Körner^1,2,3,4^, Adina Coroiu^1^, Laura Copeland^1^, Carlos Gomez-Garibello^1^, Cornelia Albani^5^, Markus Zenger^5,6*^, Elmar Brähler^5,7^

1 Department of Educational and Counselling Psychology, McGill University, Montreal, Canada, 2 Department of Oncology, McGill University, Montreal, Canada, 3 Louise-Granofsky-Psychosocial Oncology Program, Segal Cancer Centre, Jewish General Hospital, Montreal, Canada, 4 Psychosocial Oncology Program, McGill University Health Centre, Montreal, Canada, 5 University of Leipzig, Department of Medical Psychology and Medical Sociology, Leipzig, Germany, 6 Faculty of Applied Human Studies, University of Applied Sciences Magdeburg and Stendal, Stendal, Germany, 7 Department of Psychosomatic Medicine and Psychotherapy, Universal Medical Center Mainz, Mainz, Germany.
